# Feeding Faba Beans (*Vicia faba* L.) Reduces Myocyte Metabolic Activity in Grass Carp (*Ctenopharyngodon idellus*)

**DOI:** 10.3389/fphys.2020.00391

**Published:** 2020-04-24

**Authors:** Jing-jing Tian, Bing Fu, Er-meng Yu, Yu-ping Li, Yun Xia, Zhi-fei Li, Kai Zhang, Wang-bao Gong, De-guang Yu, Guang-jun Wang, Jun Xie

**Affiliations:** Key Laboratory of Tropical & Subtropical Fishery Resource Application & Cultivation, Pearl River Fisheries Research Institute, Chinese Academy of Fishery Sciences, Guangzhou, China

**Keywords:** crisp grass carp, energy status, faba bean, myocyte development, RNA-seq

## Abstract

In this study, we aimed to explore the effects of faba bean (*Vicia faba* L.) on the energy metabolism of grass carp (*Ctenopharyngodon idellus*). A total of 180 fish (∼2900 g) were randomly assigned to six tanks (2.5 × 2.5 × 1.2 m; 30 individuals per tank) and fed either faba bean (*Vicia faba* L.) or a commercial diet for 120 days (3% body weight, twice per day). The results showed that faba bean-fed grass carp (FBFG) had significantly lower growth and higher fat accumulation in the mesenteric adipose tissue and hepatopancreas than commercial diet-fed grass carp (CDFG). Compared with CDFG, FBFG exhibited no significant difference in proximate composition of the muscle; however, an obvious decrease in muscle fiber size and significantly higher hardness, chewiness, and gumminess were observed. Transcriptome results showed that a total of 197 genes were differentially regulated in the dorsal muscle. Down-regulated genes included four genes annotated with myocyte development and 12 transcripts annotated with components of myofibrils. In addition, the FBFG group exhibited significantly lower expression of genes associated with oxygen transport, the mitochondrial respiratory chain, and creatine metabolism, suggesting reduced energy availability in the muscle of the FBFG. Moreover, using western-blotting and enzyme assays, we found decreased protein levels in the mitochondrial electron transport respiratory chain and creatine metabolism activities, as well as increased expression of autophagy marker protein levels, in the muscle of FBFG. Overall, our results suggest that an abnormal energy distribution may exist in grass carps after feeding with faba bean, which is reflected by a mass of fat deposition in the adipose tissue and hepatopancreas and subdued metabolic activity in the muscle.

## Introduction

The aquaculture industry continues to develop its role in providing sustainable high-quality protein and lipids for consumers. In China, freshwater aquaculture occupies a large proportion of total aquaculture production. Approximately 29.60 million tons of freshwater products were produced in China in 2018, of which grass carp was the most cultured (18.60%, 5.50 million tons) (Fisheries Bureau of Ministry of Agriculture, 2019). In the last decades, faba bean-fed grass carp (FBFG) has gradually taken a role as a flagship fish of sorts, not only welcomed in China, but also United States and various countries in Southeast Asia and Latin America (Yang et al., 2015). FBFGs display increased muscle hardness and crispness after they are fed faba bean for 90 to 120 days (Tian et al., 2019). Compared with commercial diet fed-grass carp (CDFG), FBFG needs a strict culture environment of fresh water with high dissolved oxygen (Xie et al., 2012). Moreover, FBFG presents lower growth and swimming motility, as well as higher mortality than CDFG (Yu et al., 2017; Tian et al., 2019). Interestingly, faba bean readily induces fat accumulation in the visceral tissues (such as the adipose tissue and hepatopancreas) compared with commercial diets, suggesting an unreleased energy status in FBFG. This phenomenon has a large probability of impacting FBFG muscle development, and may be the reason for lower growth and swimming motility, and even other changes in muscle features (Tian et al., 2019).

The locomotory muscle of fish is the main edible part consumed by humans (Altringham and Ellerby, 1999). Muscle growth is the result of both the recruitment of new myocytes (hyperplasia) and hypertrophy of existing myocytes (Alami-Durante et al., 2018). It is suggested that muscle hyperplasia is maintained during a large part of the life cycle in fish species that reach a large adult body size, which is different from that in other vertebrate groups (Rowlerson and Veggetti, 2001). Although poorly understood in fish, the steps of myogenesis in mouse models provide clues, including commitment of muscle precursors, myoblast proliferation, migration, alignment, and fusion into myotubes (Buckingham and Rigby, 2014). Myocytes are mainly filled with myofibrils, which are long protein cords composed of myofilaments comprising molecules called myosin, actin, or titin (Sanger et al., 2016; Robison and Prosser, 2017). The relative contribution of hyperplasia and hypertrophy to muscle growth is known to be regulated by many nutritional factors, among which, proteins play a major role, given the importance of protein deposition on muscle growth (Alami-Durante et al., 2018). However, emerging evidence has suggested that bioenergetic pathways are intimately linked to myocyte differentiation, especially mitochondrial biology (Porter et al., 2011). In the aquaculture industry, there is evidence indicating that moderate and sustained exercise at optimal speeds can improve growth and feed conversion in some fish species (Palstra and Planas, 2011; Vélez et al., 2017). An increase in dietary lipid level or lipolytic efficiency also presented a protein-sparing effect in many fish species, such as the blunt snout bream (Li et al., 2010), sea bream (Company et al., 1999), and grass carp (Gao et al., 2011; Tian et al., 2015; Shi et al., 2018). On the contrary, energy shortage decreases growth and induces autophagy (Singh and Cuervo, 2011; Peng et al., 2017; Wang et al., 2018).

In organisms, including fish, energy transduction is mainly in the form of ATP and the energy status positively depends on ATP production. Among all the organic molecules, lipids are suggested to play an important role in ATP production in fish (Sargent et al., 2003). Fatty acids can be transported into the mitochondria for β-oxidation by a series of enzymes to produce the end-product Acetyl-CoA (Bartlett and Eaton, 2004). Acetyl-CoA then enters the tricarboxylic acid cycle to form NADPH and FADH_2_, which are absorbed by the mitochondrial respiratory chain, where five enzymatic complexes produce ATP. During oxidative phosphorylation, an oxygen molecule is needed to accept electrons (Tocher, 2003; Akram, 2014). Thus, lipid supply may determine ATP production and further influence the energy status of the whole body. On the contrary, muscle is an important site for glucose storage and lipid utilization (Badin et al., 2013). Lipids are stored as triacylglycerols (TAG) within the muscle, but these TAG stores represent a small fraction of muscle lipid stores (Badin et al., 2013). Interestingly, the lipid fuel used by skeletal muscle during contractile activity is largely derived from sources outside the muscle, as suggested by the results showing that swimming training enhances the lipid content and lipoprotein lipase of muscles and decreases free fatty acids from plasma in fish (Anttila et al., 2010; Magnoni et al., 2014). Thus, considering that large energy was “locked” in the visceral tissues, we hypothesize that the energy of muscles is diminished, leading to reduced myocyte development in FBFG. Hence, the purpose of this study was to investigate lipid distribution, energy status, and metabolic activity of muscles in faba bean-fed grass carps.

## Materials and Methods

### Experimental Procedures

Grass carps were obtained from a commercial farm in Guangzhou (Guangdong, China). Feeding experiments were carried out at the Pearl River Fisheries Research Institute. Fish were reared in concrete tanks and fed with a commercial diet (gross energy 17.24 MJ/kg; moisture 9.21%, crude protein 29.62%, crude lipid 3.56%, crude ash 7.83%, and crude fiber 11.62%, DM) for two weeks to allow acclimatization to the experimental environment. The commercial diet contained: fish meal, 5 g kg^–1^; soybean meal, 215 g kg^–1^; cottonseed meal, 80 g kg^–1^; rapeseed meal, 200 g kg^–1^; wheat flour, 180 g kg^–1^; rice bran, 150 g kg^–1^; lees powder, 50 g kg^–1^; malt root, 50 g kg^–1^; choline chloride, 20 g kg^–1^; mineral mixture 20 g kg^–1^; vitamin mixture, 30 g kg^–1^. Before feeding experiments, fish were fasted for 24 h. A total of 180 fish (approximately 2900 g body weight) were randomly assigned to six tanks (2.5 × 2.5 × 1.2 m; 30 individuals per tank). Three tanks were randomly assigned to one of two groups: the control group was fed with the commercial diet; the treatment group was fed with faba beans (gross energy 15.87 MJ/kg; moisture 11.79%, crude protein 27.48%, crude lipid 0.75%, crude ash 2.60%, and crude fiber 8.42%, DM). The feeding period was 120 days. Faba beans were purchased from a local market in Guangzhou. Based on the experience of the industry, these faba beans were soaked in a saline solution for 24 h before feeding, to soften the diets and make them easily absorbable. Fish were fed with 3% their body weight twice per day (at 8:00 and 16:00). To maintain water quality, one-third of the water was renewed daily during feeding. The water temperature was maintained between 27 and 31°C. Dissolved oxygen was maintained approximately at saturation (7 mg L^–1^) through continuous aeration. The photoperiod was held constant at 12 h light/12 h dark.

### Sampling Procedures

After feeding for 120 days, all fish were weighed, and eight fish from each tank were randomly sampled. Before sampling, the fish were fasted for 24 h and then anesthetized with tricaine methanesulfonate (MS222). Two fish from each tank were selected for blood collection from the caudal vein; then, the blood was left to clot at 4°C for 4 h, and subsequently centrifugated to obtain the serum. The remaining fish were killed and dissected. The viscera, hepatopancreas and intraperitoneal fat (IPF) were removed and weighed. The hepatopancreas was then fixed in a paraformaldehyde solution for histological analyses and stored in liquid nitrogen at −80°C for further analysis. Visceral index (VI), hepatopancreas index (HI), and intraperitoneal fat index (IPFI) were calculated using the following formulae: VI (%) = viscera weight × 100/body weight; HI (%) = hepatopancreas weight × 100/body weight; IPFI (%) = IPF weight × 100/body weight.

Texture analysis and proximate analysis were conducted on dorsal muscle samples from three fish from each tank (20 × 20 × 20 mm). The remaining samples from the same three fish were also fixed in a paraformaldehyde solution for histological analyses. Samples of the dorsal muscle from the three fish remaining per tank (9 fish per group) were frozen in liquid nitrogen and then stored at −80°C for RNA extraction, protein isolation, and enzyme and metabolite analysis.

### Histological Analyses of Hepatopancreas

The hepatopancreas were fixed in 4% paraformaldehyde overnight at 4°C and incubated with 30% sucrose at 4°C for 3 days. Then, the hepatopancreas were embedded in optimal cutting temperature (OCT) compound (Leica, Germany), sectioned at 6–10 μm and rinsed with distilled water. Slides were permeated in 60% isopropanol for 20–30 s and stained with Oil red O (Sigma, United States) for 10 min. Slides were immediately destained in 60% isopropanol for 3 min and washed with distilled water to clean the background. Then the sections were counterstained with Mayer’s hematoxylin for 1 min, and washed with distilled water for 10 min. Afterward, the slides were seal-capped with glycerogelatin. The slides were photographed using a light microscope (Olympus BX41, Japan).

### Alanine Aminotransferase (ALT) and Aspartate Aminotransferase (AST) Assays

Serum ALT and AST activities were measured using assay kits (Jiancheng Biotech Co.). These two indexes were measured based on the user manual. All assays were performed in triplicate for each tank.

### Proximate Composition of Dorsal Muscles

The crude composition of diets and tissues was determined according to the Association of Official Analytical Chemists [AOAC] (1995) procedures. In summary, samples were dried to a constant weight to determine moisture content at 105°C. Crude protein content was determined by measuring nitrogen (N × 6.25) in the samples using the Kjeldahl method. Crude lipid content was measured by ether extraction using the Soxhlet method. Crude ash was determined by combustion at 550°C in a muffle furnace.

### Hematoxylin and Eosin (H&E) Muscle Staining

The samples of fixed muscle were washed in tap water for 12 h, followed by a routine dehydration in a graded series of ethanol (30, 50, 70, 80, 90, 95, and 100% twice). Samples were then equilibrated in xylene and embedded in paraffin based on standard histological techniques previously described (Yu et al., 2017). Afterward, 5-μm sections were cut with a rotary microtome (RM2235, Leica, Germany), mounted on glass slides, and stained with H&E. Histological samples were observed and photographed using an upright microscope (Leica biosystems, Wetzlar, Germany). The average myocyte size per image was quantified using Photoshop (Adobe) as previously described (Tian et al., 2017). An average value across three non-overlapping images (three/section) was calculated for each group.

### Texture Analyses of the Muscles

The texture of the dorsal muscles, including hardness, chewiness, and gumminess, was determined with a CT3 Texture Analyzer (Brookfield Engineering Laboratories, Inc., Brookfield, United States). A portion of grass carp back muscle (at the junction of the fifth dorsal fin and the lateral line scales) was collected. A P35 cylindrical probe of the CT3 Texture Analyzer was used to test the compression speed at a pre-test speed of 2 mm s^–1^, a post-test speed of 5 mm s^–1^, and a test speed of 1 mm s^–1^. The compression interval was 2 s, with a compression ratio of 25%. Three samples from each tank were prepared, and each sample was tested three times.

### RNA Extraction, Transcriptome Library Preparation, and Illumina Sequencing

The dorsal muscles from three fish per tank were used to extract total RNA using RNAiso Plus (TaKaRa, Dalian, P.R. China) according to the manufacturer’s instructions. Concentration and purity of RNA were determined by the ratio of A_260_ to A_280_ (A_260__:__280_ ≥ 1.8 and ≤ 2.0) using a NanoDrop ND-1000 spectrophotometer (Nanodrop Technologies, Wilmington, DE, United States). RNA integrity (RIN ≥ 7 and 28S/18S ≥ 0.7) was assessed on an Agilent 2100 Bioanalyzer Lab-on-chip system (Agilent Technologies, Palo Alto, CA, United States). Equivalent amounts of total RNA from the three samples were combined in each tank; thus, each treatment resulted in three pooled datasets. The total RNA samples were first treated with DNase I to degrade any possible DNA contamination. Then, the mRNA was enriched by using the magnetic beads with Oligo (dT). Mixed with the fragmentation buffer, the mRNA was fragmented into short pieces. Next, the first strand of cDNA was synthesized by using random hexamer-primer. Buffer, dNTPs, RNase H, and DNA polymerase I were added to synthesize the second strand. The cDNA fragments were purified with magnetic beads. End reparation and 3’-end single nucleotide A (adenine) addition was then performed. Finally, sequencing adaptors were ligated to the fragments. The fragments were enriched by PCR amplification. During the QC step, Agilent 2100 Bioanalyzer and ABI StepOnePlus Real-Time PCR System were used for qualification and quantification of the sample library. The library products were ready for sequencing via Illumina HiSeq^TM^ 2000.

### Transcriptome Assembly, Identification of Differentially Expressed Genes (DEGs), and Annotation

Raw reads were cleaned by removing reads containing adapters, reads with over 10% unknown nucleotides ‘N,’ and low-quality reads containing more than 50% low-quality (Q-value ≤ 5) bases. These reads were then mapped to the grass carp genome with *BWA* (Li and Durbin, 2009). The metric “fragments per kilobase of exon per million mapped reads” (FPKM) was employed to quantify gene expression by ***RSEM*** (Li and Dewey, 2011). Based on the FPKM results, the correlation value between each sample pair was calculated. The DEGs were selected based on the expression profiles and the following criteria: the change in gene expression levels in CDFG versus FBFG was | log_2_ ratio| > 1 and *False Discovery Rate (FDR)* ≤ 0.001.

Finally, the obtained DEGs were annotated against the Gene Ontology (GO) and Kyoto Encyclopedia of Genes and Genomes (KEGG) using BLAST with a cut-off e-value of 0.00001. GO was developed to represent common and basic biological information in annotation (Ye et al., 2006; Conesa and Götz, 2008). GO enrichment analysis provides all GO terms that are significantly enriched in DEGs compared to the genome background, and filters the DEGs that correspond to biological functions. To accomplish this, first, all DEGs were mapped to GO terms in the Gene Ontology database (http://www.geneontology.org/). Then, gene numbers were calculated for every term, and significantly enriched GO terms in DEGs compared to the genome background were defined by hypergeometric tests. Pathway enrichment analysis identified a significantly enriched metabolic pathway or signal transduction pathways in DEGs compared with the whole genome background (Kanehisa and Goto, 2000; Altermann and Klaenhammer, 2005).

### Quantitative Real-time PCR

To validate the results of the DEG analyses, eight functionally interesting unigenes, including four myocyte development genes, and four energy metabolism genes were chosen and evaluated by qRT-PCR. The specific primers are shown in Supplementary Table S1. The total RNA of the sampled muscles from nine fish per group was extracted according to the method described above. Next, cDNA was synthesized with PrimeScript^®^ RT reagent kit (TaKaRa, Dalian, P.R. China) following the manufacturer’s protocol. Real-time qRT-PCR was performed in triplicate (CFX 96 Real-Time PCR Detection System, Bio-Rad, California, United States) in a final volume of 20 μL containing: 0.6 μL of each primer (0.5 μM), 1 μL diluted first strand cDNA product, 10 μL 2 × SYBR Premix Ex Taq II (TaKaRa, Dalian, P.R. China), and 7.8 μL sterilized double-distilled water. The thermocycling parameters were 95°C for 30 s, followed by 40 cycles of 95°C for 15 s, and 60°C for 15 s. After PCR, the melting curve was analyzed over a range of 60–95°C (in 5 s steps) to confirm a single product. To ensure that only the cDNA was quantified in each sample, negative controls included a no-cDNA control and a DNase-treated non-reverse transcribed tissue RNA sample. The β-actin gene (GenBank accession No. DO211096) was used as the reference gene based on preliminary tests using geNorm (version 3.5) and NormFinder algorithms. A relative quantification method was used to calculate the gene expression values using the comparative CT method (2^–ΔΔCt^) previously described in the literature (Livak and Schmittgen, 2001; Pfaffl, 2001).

### Western Blot Analysis

Dorsal muscle from five FBFGs and CDFGs were homogenized with glass Tenbroeck tissue grinders on ice. Cell lysis buffer supplemented with protease and phosphatase inhibitor cocktails (Roche) was added before homogenization. Next, the crude lysates were centrifuged at 4°C for 10 min at 13, 000 × *g*, after which the upper mixture from each lysate was separated to be used for further analysis. Total protein concentration from the resultant supernatant was determined by a bicinchoninic acid protein assay kit (Thermo Fisher Scientific Inc., United States). Protein samples were separated by SDS-PAGE and transferred to a polyvinylidene difluoride membrane (Beyotime Technologies Inc.) by electroblotting. Membranes were incubated overnight at 4°C with primary antibodies. After washing, the secondary antibody (Beyotime Technologies Inc.) was added and incubated for 2 h at room temperature and the protein bands were visualized by ECL Plus (ZETA). The membranes were then stripped and reprobed with anti-GAPDH antibody. Densitometric quantitation was performed using a Sagecreation imaging system with Sagecreation Quantity One software (Sagecreation Co., Ltd.). The following antibodies (Abcam, Cambridge, MA) were used: antibodies against anti-MT-CO1 (40 kDa; mouse), anti-MT-CO2 (26 kDa; mouse), anti-MT-ND1 (36 kDa; rabbit), anti-complex III-UQCRC1 (53 kDa; rabbit), anti-PGC1 (92 kDa; rabbit), anti-Bnip3 (60 kDa; rabbit), anti-Parkin (55 kDa; mouse), and anti-LC3B (17,19 kDa; rabbit).

### Creatine Kinase Activity and Creatine Level Assay

Dorsal muscle from five FBFGs and CDFGs were homogenized with glass Tenbroeck tissue grinders on ice. The creatine kinase activity (Jiancheng Biotech Co., Nanjing, China) and creatine level (BioAssay Systems, CA, United States) of the muscles were assayed using the kits according to the manufacturer’s instructions.

### Statistical Analyses

The data are expressed as means ± S.D. (standard deviation). Percentage data were arcsine-transformed prior to analysis. Differences were determined using an independent-samples *t*-test. All analyses were conducted using PASW Statistics 18 (SPSS, Chicago, IL, United States). Statistical significances are denoted with asterisks as follows: ^∗^*P* < 0.05; ^∗∗^*P* < 0.01; ^∗∗∗^*P* < 0.001.

## Results

### Biological Parameters, Fat Accumulation in Grass Carps Fed With Faba Bean

**FIGURE 1:**
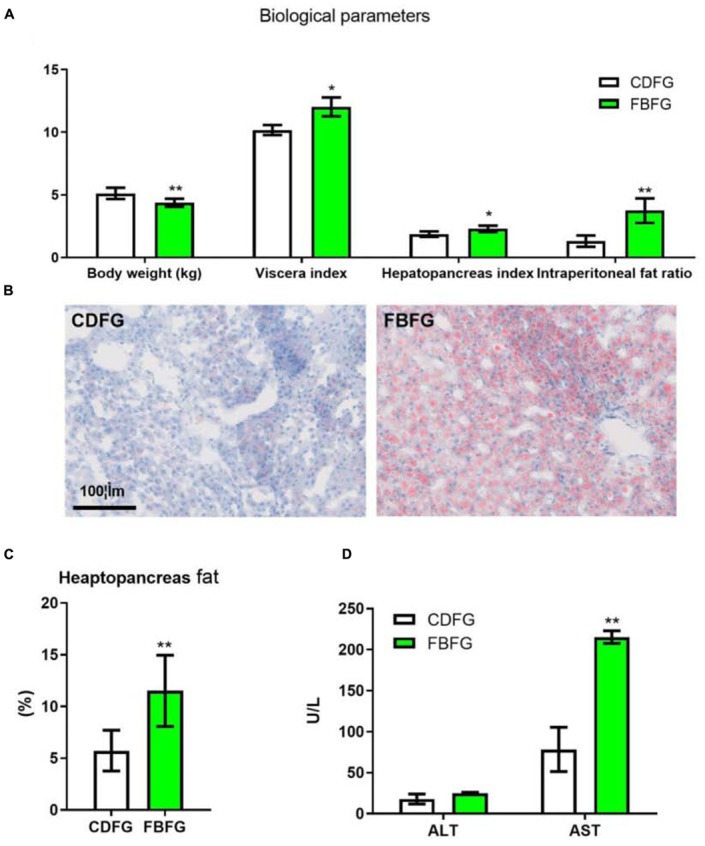
Biological parameters **(A)**, hepatopancreas histology **(B)**, fat content **(C)**, serum ALT, and AST activities **(D)** in grass carp fed with commercial diet (CDFG) or faba bean (FBFG) for 120 days. All results are presented as mean ± SD (SD is represented by error bars; *n* = 3). Statistical significance is denoted with asterisks as follows: *P < 0.05; **P < 0.01; ***P < 0.001. ALT, alanine aminotransferase; AST, aspartate aminotransferase; CDFG, commercial diet-fed grass carp; FBFG, faba bean-fed grass carp. Acronyms used throughout.

After feeding with faba bean for 120 days, the body weight of FBFGs was significantly lower than that of CDFGs. However, FBFGs showed significantly higher visceral index, hepatopancreas index, and intraperitoneal fat ratio than CDFGs (Figure 1A; *P* < 0.05). Moreover, FBFGs presented more lipid droplets and fat content in the hepatocytes (Figures 1B,C; *P* < 0.01). In addition, serum AST activities in FBFGs significantly increased compared with that in CDFGs (Figure 1D; *P* < 0.01).

### Composition, Morphology, and Texture of the Muscle in Grass Carps Fed With Faba Bean

**FIGURE 2:**
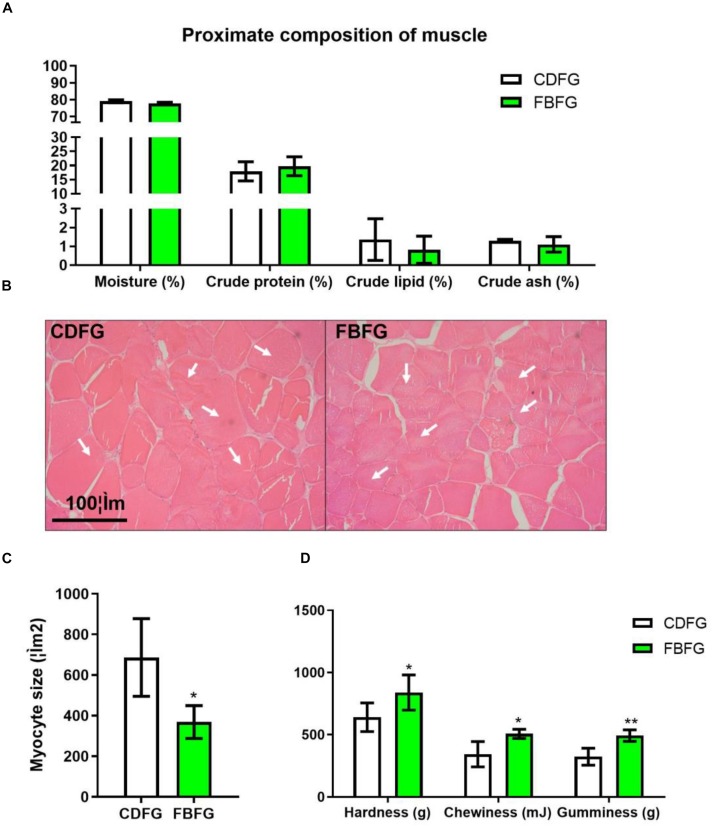
Proximate composition **(A)**, histology **(B)**, myocyte size **(C)**, and TPA value **(D)** of dorsal muscle in grass carp fed with commercial diet (CDFG) or faba bean (FBFG) for 120 days. All results are presented as mean ± SD (SD is represented by error bars; *n* = 3). Statistical significance is denoted with asterisks as follows: *P < 0.05; **P < 0.01; ***P < 0.001.

In this study, no obvious differences were observed in the proximate composition of muscles, such as moisture, crude protein, crude lipid, and crude ash, between the two groups (Figure 2A). However, FBFGs exhibited a significant decrease in muscle fiber size compared with CDFG as shown by H&E staining (Figures 2B,C). Texture indexes, including hardness, chewiness, and gumminess, were all significantly increased in the dorsal muscle of FBFGs (Figure 2D).

### Relative mRNA Expression Profiling and DEGs of the Muscle

To obtain an overview of the muscle gene expression profile of grass carps in response to different diets, six cDNA samples were generated from these two groups and sequenced. A total of 78,750,308 raw reads (3.59 G of data bulk) were generated (Table 1). After filtering the low-quality reads, a total of 78,712,282 high-quality clean reads with 3,856,901,818 bp were obtained. We then matched these sequences to the reference genome and more than 76% reads were mapped, of which no less than 16,871 and 15,462 genes for CDFGs and FBFGs were identified, respectively (Table 1).

**TABLE 1:** Summary of the transcriptome in dorsal muscle of grass carp.

	**CDFG1**	**CDFG 2**	**CDFG 3**	**FBFG1**	**FBFG 2**	**FBFG 3**
Raw reads	13,124,981	13,124,852	13,125,561	13,124,574	13,125,181	13,125,159
Clean reads	13,115,472	13,117,849	13,118,664	13,119,853	13,119,933	13,120,511
Total base pairs (bp)	642,658,128	642,774,601	642,814,536	642,872,797	642,876,717	642,905,039
Total mapped reads	10,091,440	10,134,652	10,108,835	10,047,431	10,067,025	10,022,343
	(76.94%)	(77.26%)	(77.06%)	(76.58%)	(76.73%)	(76.39%)
Expressed gene	16,871	17,608	16,884	15,635	15,608	15,462

**FIGURE 3:**
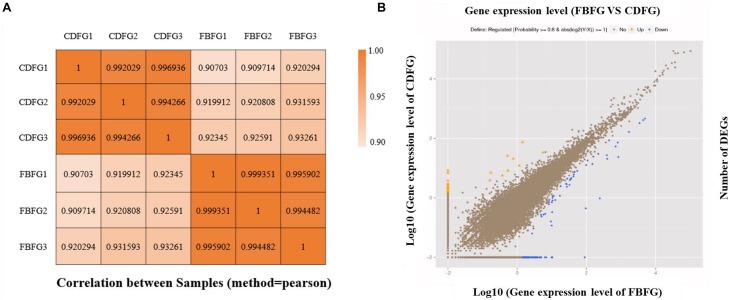
Correlation heatmap **(A)** and scatter **(B)** diagram of differentially expressed genes (DEGs) of dorsal muscle in grass carp fed with commercial diet (CDFG) or faba bean (FBFG) for 120 days. Yellow dots mean high expression in FBFG compared with CDFG and blue dots mean low expression in FBFG compared with CDFG.

To calculate the gene expression levels, the metric FPKM was calculated using *RSEM*. Based on the FPKM results, the correlations among tanks were then evaluated. The Pearson correlation coefficients were approximately 0.92 between fish fed with the commercial diet and those fed with faba bean, whereas they were no less than 0.99 among the same treatments (Figure 3A), suggesting a substantive difference between the fish fed with the two kinds of diets. Based on the criteria of two-fold or greater change and *FDR* < 0.001, a total of 197 DEGs were selected, among which 33 were up-regulated and 164 were down-regulated (Figure 3B).

To gain insights into the biological processes affected by the different diets, and identify the processes enriched in DEGs, we subjected DEGs to GO term enrichment analysis. In this study, 160 GO term annotations of DEGs were produced and assigned to three categories (Supplementary Figure S1). Among them, a total of 34, 44, and 48 DEGs were assigned to Cellular Components, Molecular Functions, and Biological Processes, respectively. DEGs that were most enriched in GO biological process terms were metabolic processes (25), single-organism processes (24), and cellular processes (21). To further appreciate the biological functions of DEGs, a KEGG pathway analysis was used. The results showed that 118 DEGs were assigned to 148 KEGG pathways. Among them, influenza A (18), NOD-like receptor signaling pathway (16), and pertussis (16) were the most frequently represented (Supplementary Figure S2).

### Expression Pattern of Myocyte Development Related Genes

Based on the annotation, we selected the DEGs that were annotated with genes related to myocyte development (Table 2). On the one hand, four DEGs, annotated with myocyte proliferation, *nicotinamide riboside kinase 2 (NRK2), proheparin-binding egf-like growth factor isoform X1(HB-EGF), AMP deaminase (AMPD)*, and *myotubularin-related protein 3-like (Mtmr3)*, were significantly down-regulated in FBFGs. On the other hand, a total of 12 transcripts annotated with proteins that are components of muscle fiber, such as *tubulin beta-1 chain (TUBB1)* and *myosin heavy chain b (Myhb)*, were down-regulated in the muscles of FBFGs (Table 2).

**TABLE 2:** Representative DEGs related to myocyte development, myofibrils components, oxygen transportation, mitochondrial respiratory chain, creatine metabolism, and cell activity of the muscle in grass carp in response to faba bean.

**Gene ID**	**Gene description**	**log_2_Ratio(FC)**
***Myocyte development***		
CI01000030_10982784_10985399	Nicotinamide riboside kinase 2 (NRK2)	–3.5445
CI01000006_02227697_02229505	Proheparin-binding EFG-like growth factor isoform X1 (HB-EGF)	–7.84758
CI01000185_00000012_00003634	AMP deaminase (AMPD)	–3.39968
CI01153174_00000505_00001049	Myotubularin-related protein 3-like (Mtmr3)	–7.78572
***Myofibrils components***		
CI01000120_00561737_00562665	Troponin I type 2a (skeletal, fast), tandem duplicate 1 (TNNI2A1)	–4.28529
CI01023542_00000355_00000744	Myosin heavy chain, fast skeletal muscle-like (Myhfs)	–2.95826
CI01020178_00000113_00000322	Arf-gap with rho-gap domain, ANK repeat and PH domain-containing protein 1-like (Arap1)	–7.51043
CI01000021_03428718_03432817	Krt5 protein (Krt5)	–8.78681
CI01000018_03727066_03727226	LIM domain and actin-binding protein 1-like isoform X3 (LIMA1 × 3)	–7.57238
CI01000340_14509233_14513910	Tubulin beta-1 chain (TUBB1)	–3.8194
CI01000047_06293132_06300073	Ras-related C3 botulinum toxin substrate 3 (rho family, small GTP binding protein rac3) (RAC3)	–7.25739
CI01000193_00088221_00095804	Transglutaminase 2 (TGM2)	–3.51166
CI01000299_00005753_00010092	Interferon-induced protein 44 (IFI44)	–8.84235
CI01000030_07307842_07309188	Mid1-interacting protein 1 (MIDIP1)	–3.44231
CI01143068_00000078_00000628	Myosin heavy chain b (Myhb)	–7.97985
CI01000016_04815074_04817608	Novel protein similar to vertebrate titin (TTN)	–8.49586
***Oxygen transportation***		
CI01000113_00332256_00333023	Novel alpha globin (NHBA)	–3.71153
CI01000113_00285871_00286497	Hemoglobin beta chain (HBB)	–3.46213
CI01000113_00324040_00324756	Alpha globin (HBA)	–3.406
CI01000113_00326077_00326875	Beta globin (HBB)	–3.41766
CI01000034_03869116_03883535	Solute carrier family 4, anion exchanger, member 1 (Slc4a1)	–3.12988
***Mitochondrial respiratory chain***		
CI01000304_17694317_17697897	Ucp2l protein (UCP2l)	–2.75296
CI01000009_01966898_01976924	Coenzyme q-binding protein COQ10 homolog B, mitochondrial precursor (COQ10B)	–2.58322
CI01000325_05626291_05627850	Cytochrome c oxidase subunit 8A, mitochondrial-like (COX8A)	–8.43741
CI01000004_10290550_10295288	Carbonic anhydrase (CA)	–2.70116
***Creatine metabolism***		
CI01000166_00646051_00651860	Glycine amidinotransferase, mitochondrial (GATM)	–5.1498
CI01000010_08642328_08681400	Sodium- and chloride-dependent creatine transporter 1 isoform X1 (SCCT1)	–3.04809
***Cell activity***		
CI01000010_01954571_01961365	Sodium-dependent phosphate transporter 1-A isoform X1 (SPT1)	–3.07552
CI01000319_05746833_05748261	ATP-binding cassette, sub-family B (MDR/TAP), member 3, like 2 (ABCB3)	–8.2384
CI01000086_01909580_01912644	Krueppel-like factor 10 (KLF10)	–2.82561

### Expression Pattern of Energy Metabolism Related Genes

Genes that were annotated with energy metabolism were also influenced by the different diets (Table 2). First, a total of seven DEGs annotated with oxygen transport (including *alpha globin [HBA] and hemoglobin beta chain [HBB]*) were down-regulated in FBFGs. Second, the expression of four DEGs that were related to mitochondrial respiratory chain, such as *UCP2l protein (UCP2l)* and *Cytochrome c oxidase subunit 8A (COX8A)*, were down-regulated in response to the faba bean diet. Third, two genes annotated with creatine metabolism, *Glycine amidinotransferase (GATM)* and *Sodium- and chloride-dependent creatine transporter 1 isoform X1 (SCCT1)* were down-regulated in FBFGs. Finally, three cell activity marker genes, such as *Sodium-dependent phosphate transporter 1-A isoform X1 (SPT1)* and *ATP-binding cassette, sub-family B (MDR/TAP), member 3, like 2 (ABCB3)* were also down-regulated in grass carps fed with faba bean (Table 2).

### Validation of DEGs From Transcriptomes

**FIGURE 4:**
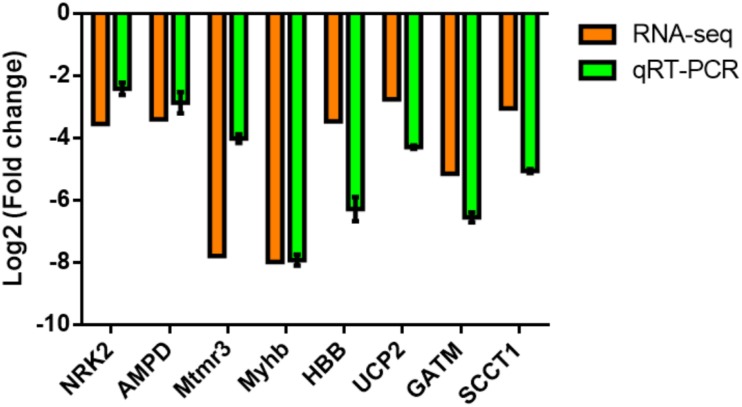
Comparison of the expression levels of RNA-Seq and qRT-PCR results in the dorsal muscle of grass carp fed with commercial diet (CDFG) or faba bean (FBFG) for 120 days. Transcript levels of the selected genes are normalized to that of the β-actin gene.

To validate the reliability of transcriptome, eight interesting functional genes, including four myocyte development (*NRK2*, *AMPD*, *Mtmr3*, and *Myh*b) and four energy metabolism (*HBB*, *UCP2*, *GATM*, and *SCCT1*) annotated transcripts were selected for quantitative real time-PCR (qRT-PCR) analysis. The qRT-PCR results showed that all of these genes were consistent with those obtained from the transcriptome sequencing (Figure 4).

### Expression of Proteins Related to Energy Metabolism

**FIGURE 5:**
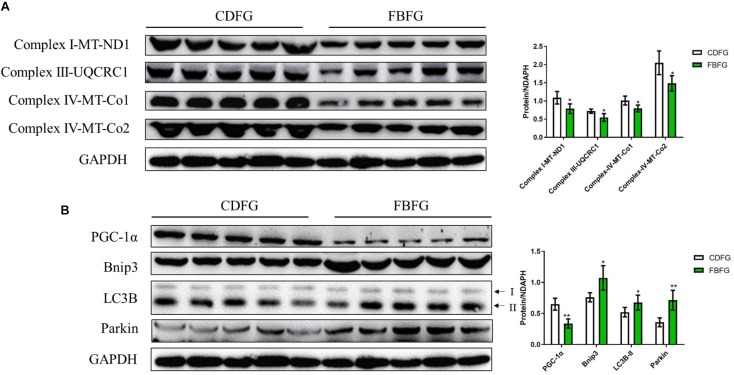
Comparison of the protein expression levels of the mitochondrial electron transport respiratory chain **(A)**, mitochondria development and autophagy **(B)** in the muscle between commercial diet-fed grass carp (CDFG) and faba bean-fed grass carp (FBFG). Results are presented as mean ± SD (SD is represented by error bars; *n* = 5). Statistical significance is denoted with asterisks as follows: *P < 0.05; **P < 0.01; ***P < 0.001. ND1, NADH dehydrogenase 1; UQCRC1, ubiquinol-cytochrome c reductase core protein 1; Co, cytochrome c oxidase; PGC-1α, peroxisome proliferator-activated receptor-γ coactivator 1α; LC3B, microtubule-associated protein 1 light chain-3B, acronyms used throughout.

**FIGURE 6:**
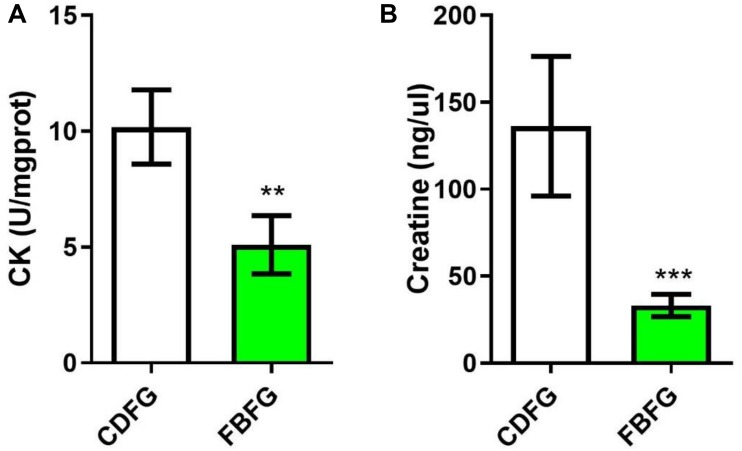
Comparison of the creatine kinase (CK) activity **(A)** and creatine level **(B)** in the muscle between commercial diet-fed grass carp (CDFG) and faba bean-fed grass carp (FBFG). Results are presented as mean ± SD (SD is represented by error bars; *n* = 5). Statistical significance is denoted with asterisks as follows: **P < 0.01; ***P < 0.001.

To further investigate if the energy status of FBFGs was influenced by the faba bean diet, the protein levels of the mitochondrial electron transport respiratory chain were measured by western-blot. As shown in Figure 5A, marker proteins of the mitochondrial electron transport respiratory chain, such as NADH dehydrogenase 1 (a subunit of complex I), ubiquinol cytochrome c reductase core protein I (UQCRC1, a component of the complex III), cytochrome *c* oxidase 1 and cytochrome *c* oxidase 2 (Co1 and Co2; two subunits of complex IV) were significantly lower in FBFGs than in CDFGs. Furthermore, the peroxisome proliferator-activated receptor-γ coactivator 1α (PGC-1α), which has a pivotal role in mitochondrial biogenesis, was reduced in FBFGs (Figure 6B). Interestingly, two autophagy marker proteins, Bnip3 (responsible for vesicle elongation and autophagosome formation) and microtubule-associated protein 1 light chain (LC3B; functions in autophagy substrate selection and autophagosome biogenesis) were significantly higher in FBFGs than in CDFGs (Figure 5B). Finally, protein expression of Parkin (also known as Park2), a protein specifically involved in mitochondrial clearance, was also increased in FBFGs (Figure 5B).

### Creatine Metabolism Pattern of the Muscles in Grass Carps Fed With Two Different Diets

The transcriptome results suggest that the expression of *GATM* and *SCCT1* were down-regulated, suggesting a lower metabolism capacity of creatine (a molecule that facilitates recycling of ATP). To address this, we analyzed the activity of creatine kinase and the concentration of creatine in the muscle of grass carps from the two diet groups. Indeed, our results showed that fish fed with faba bean had nearly half the creatine kinase activity (*P* < 0.01), and one third the creatine level (*P* < 0.001) of fish fed with the commercial diet (Figure 6).

## Discussion

In our feeding experiment, growth was significantly lower in FBFGs than in CDFGs, similar to the observations in common industrial practice. However, FBFGs seemed to present an “obesity” phenotype as indicated by the increased fat deposition in the hepatopancreas and adipose tissue. It is suggested that the lipid accumulation induced by faba bean is a cumulative result of increasing lipogenesis and decreasing lipid oxidation, but the internal triggers remains largely unknown (Tian et al., 2019). Interestingly, faba bean has been suggested to cause intestinal damage and induce inflammation in grass carps (Li et al., 2018), and wounds have been reported to attract fat cells to drive repair and prevent infection (Franz et al., 2018). However, further studies are needed to determine whether intestinal damage or inflammation can induce fat accumulation in peri-intestinal tissues of fish. Nevertheless, our study indeed shows that low lipid faba beans may induce excessive fat accumulation in some visceral tissues (adipose tissue and hepatopancreas), which may likely be related to the metabolic status in the muscle.

In this study, although no obvious differences in the proximate composition of muscles were found, muscle structure was significantly changed as suggested by the myocyte size and TPA values, indicating an abnormal muscle phenotype in FBFGs. We further used the transcriptome technology to study the transcript profiles of the muscles in the two diet groups. More than 3G raw reads were obtained, of which up to 99% were high-quality reads, which could ensure the accuracy of detection. Based on the criteria of two-fold or greater change and *FDR* < 0.001, a total of 33 and 164 DEGs were up-regulated and down-regulated, respectively. The RNA-seq data showed that the mRNA expression of NRK2 and HB-EGF were down-regulated in FBFG group than the CDFG group. NRK2 plays a role in the regulation of terminal myogenesis by increasing myogenin (MyoG), myogenic differentiation 1 (MyoD), and α-actin 1 (Li et al., 1999; Fletcher et al., 2017). HB-EGF is a far more potent mitogen for smooth muscle cells and myogenesis through transcriptional regulation by MyoD (Davis-Fleischer and Besner, 1998; Dao et al., 2018). Down-regulation of these two genes indicates that both hyperplasia and hypertrophy of the myocytes were possibly attenuated in FBFG. Interestingly, the mRNA expression level of AMPD (associated with symptoms of metabolic myopathy when it is deficient in skeletal muscle) (Morisaki et al., 1992) and the MTMR3 (plays an important role in maintaining muscle function) (Hnia et al., 2011), decreased in our study, indirectly suggesting a metabolic disturbance condition in FBFGs. In addition, the subdued myocyte development activity could also be demonstrated by the down-regulation of component of myofibrils annotated genes, such as *Troponin I type 2a (skeletal, fast), tandem duplicate 1 (TNNI2A1)*, *Myosin heavy chain, fast skeletal muscle-like (Myhfs)*, *Tubulin-beta 1 chain* (*TUBB1)*, and *Novel protein similar to vertebrate titin (TTN)*, suggesting reduced sources for the assembly of fibers in the myocyte as suggested by Sanger et al. (2016). In summary, our results demonstrate a subdued myocyte development status in the muscle of faba bean-fed grass carps.

In the aquaculture industry, FBFGs need a high dissolved oxygen water environment for cultivation. In our study, we show that FBFGs had decreased expression of oxygen transport transcripts, possibly reflecting a relative lower oxygen utilization capacity in their muscle, which may result in hypoxia status of the fish. Actually, hypoxia was suggested to decrease growth or myogenesis in many fish species (Johnston, 2006), such as grass carp (Gan et al., 2013), European sea bass (Pichavant et al., 2001), and black bream (Hassell et al., 2008). Mechanistically, a study in mice demonstrated that hypoxia inhibits myogenic differentiation through accelerated MyoD degradation (Di Carlo et al., 2004). In this way, our results showing down-regulation of oxygen transport genes may provide evidence for high requirements of dissolved oxygen and low myocyte development activity in FBFG.

Additionally, myogenesis is suggested to be related to the bioenergetics pathways (Porter et al., 2011). Peng et al. (2017) showed that by blocking mitochondrial development in zebrafish, less energy was produced, resulting in reduced growth rates. The hypoxia status in FBFGs could also have influenced their energy production (such as ATP), since O_2_ is an irreplaceable molecule in mitochondrial processes. Indeed, our results showed that the mRNA expression of several mitochondrial respiratory chain related proteins, such as *UCP2*, *Coenzyme q-binding protein COQ10 homolog B, mitochondrial precursor (COQ10B)*, and *COX8A*, were down-regulated. Western blot results further demonstrated that energy metabolism was attenuated in FBFGs, as suggested by the decreased expression in several marker subunits of the mitochondrial electron transport chain, such as NADH dehydrogenase 1 (ND1), UQCRC1, Co1, and Co2 in FBFGs (Woldt et al., 2013). The decreased respiratory chain activity might be partly due to the lower lipid level in the diet (Eya et al., 2013). However, we cannot exclude the relationship with large lipids deposited in the hepatopancreas and adipose tissue, which possibly induce lower energy output in the muscle (Tian et al., 2019). On the contrary, we found that the mitochondrial biogenesis marker protein, PGC-1α, was decreased in FBFGs, suggesting that mitochondrial development was reduced (Liang and Ward, 2006). Meanwhile, the decreased expression of autophagy and mitophagy marker proteins, including BNIP3, LC3B, and Parkin, suggested that mitochondrial clearance may be occurring in the myocytes of grass carps fed with faba bean (Narendra et al., 2008; Zhang and Ney, 2009). Overall, our results clearly indicated subdued mitochondrial activity in FBFGs. In fact, mitochondria are suggested to be potential regulators of myogenesis (Remels et al., 2010; Wagatsuma and Sakuma, 2013). The decreased mitochondrial activity is likely to be one of the main reasons for the contribution of lower growth and fiber number in FBFGs.

The results of the decreased index associated with creatine metabolism may provide additional evidence for an energy deficient status in grass carps after a faba bean diet. Creatine is a small molecule that plays an important role in energy metabolism, which is only known to be required for a single enzymatic reaction—that of creatine kinase—which interconverts creatine and phosphocreatine in tissues with a rapid, high demand for ATP (Brosnan and Brosnan, 2016). Creatine has been suggested to increase the expression of myosin-heavy chain and stimulate muscle-specific protein synthesis in both skeletal and cardiac chicken myotubes (Ingwall et al., 1972). In human skeletal muscle fibers, creatine was shown to increase the satellite cell number and myonuclei concentration (Olsen et al., 2006). A study in C_2_C_12_ cells has also demonstrated that creatine could enhance differentiation by activating both p38 and Akt/PKB pathways (Deldicque et al., 2007). However, the role of creatine in myogenesis of teleosts has not been reported yet. Our transcriptome results showed that two transcripts related to the creatine metabolism were decreased in FBFGs, and we further demonstrated that creatine level and creatine kinase activity were also reduced in muscles of FBFGs. Although the lower creatine metabolism capacity may also be due to the energy/nutrient deficiency, these data indirectly provide evidence for the subdued myogenic activity in FBFGs.

Though many studies have explored the molecular changes in FBFGs, the mechanism of increased “hardness” in the muscle of this type of grass carp was not fully understood (Yu et al., 2017). It was suggested that fiber density was one of the main reasons for the observed change in muscle texture, which was due to the attenuation of myocyte hyperplasia (Yu et al., 2017). Our study showed a reduction in energy metabolism activity, which may be one of the reasons for subdued myogenesis in grass carps. Interestingly, muscle texture quality was influenced when fish were exposed to starvation conditions or low energy diets (Bugeon et al., 2004; Zhao et al., 2018). Our study showed that the energy was mostly deposited in some visceral tissues (adipose tissue and hepatopancreas) of FBFGs, whereas no difference in muscle lipid content was found between the two diet groups, which could further exacerbate the energy shortage in the muscle if the energy of the myokinesis were mostly derived from sources outside the myocytes. Despite these results, it remains unclear what factors induce the energy metabolism disorders in FBFGs. We cannot conclude that muscle “hardness” in FBFGs was completely due to energy deficiency, as it has been show that the hardness, chewiness and adhesiveness of grass carps decreased after short-(15 d) or long-term (50 d) complete starvation (Xia et al., 2017). Nevertheless, the relative low energy of faba bean and the accumulated fat in adipose tissue and hepatopancreas did indeed contribute to the subdued myocyte metabolic activity of the muscles studied in our study.

**FIGURE 7:**
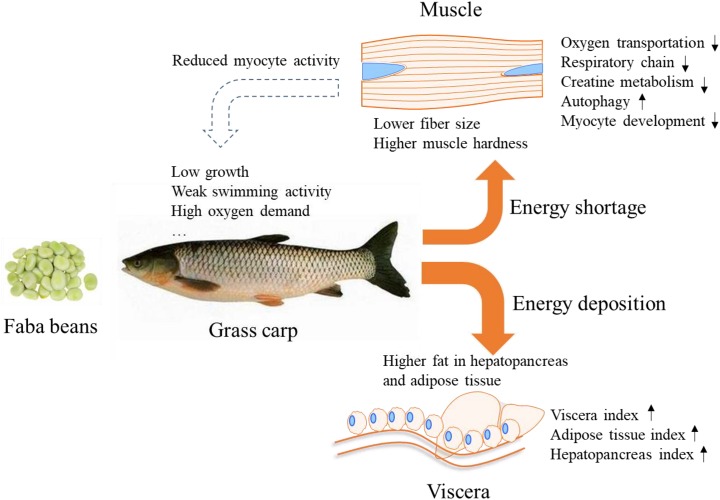
Schematic overview of the proposed energy metabolic flux of grass carp in response to faba bean. Feeding grass carp with faba bean results in most of the energy being deposited in the adipose tissue and hepatopancreas, causing energy shortage in the muscle and reduced myocyte activity. The phenomenon may be the main reason for the slow growth, weak swimming activity, and high oxygen demand reported in faba been-fed grass carp.

In conclusion, we compared the energy status of grass carps in response to either commercial diet or faba bean diet. Our data show that faba bean-fed fish deposited more fat in the hepatopancreas and mesenteric adipose tissue, and presented reduced oxygen transport and reduced mitochondrial respiratory chain and creatine metabolism capacity in muscle tissue than the commercial diet-fed fish. The subdued energy metabolic activity of myocytes should provide new references for the low growth, weak swimming activity, high mortality, and hard fillet of faba bean-fed grass carps (Figure 7).

## Ethics Statement

This study was carried out in accordance with the principles of the basic declaration and recommendations of the Institutional Animal Care and Use Committee and performed in accordance with national and institutional regulations on the care and use of experimental animals. The protocol was approved by the Institutional Animal Care and Use Ethics Committee of the Chinese Academy of Fishery Sciences.

## Data Availability Statement

The sequencing data in this study have been deposited in the Sequence Read Archive (SRA) at the National Center for Biotechnology Information (NCBI) (accession number: PRJNA548646).

## Author Contributions

JT, EY, and JX conceived and designed the experiment. JT, BF, EY, YL, and YX performed the experiment, contributed to the analysis of data and manuscript writing. ZL, KZ, GW, DY, and WG contributed to the revision of the manuscript. All authors read and approved the final manuscript.

## Conflict of Interest

The authors declare that the research was conducted in the absence of any commercial or financial relationships that could be construed as a potential conflict of interest.
