# Supplementary material for: Feeding Faba Beans (Vicia faba L.) Reduces Myocyte Metabolic Activity in Grass Carp (Ctenopharyngodon idellus)
Source: Front Physiol. 2020 Apr 24;11:391. doi: 10.3389/fphys.2020.00391 (PMC7197471; doi:10.3389/fphys.2020.00391)
Supplement: Supplementary file 1 [file Table_1.DOCX]

**Supplemental materials**

**Figure S1 Gene ontology (GO) categories of the differentially expressed genes (DEGs) of dorsal muscle in grass carp fed with commercial diets or faba beans for 120 days.**


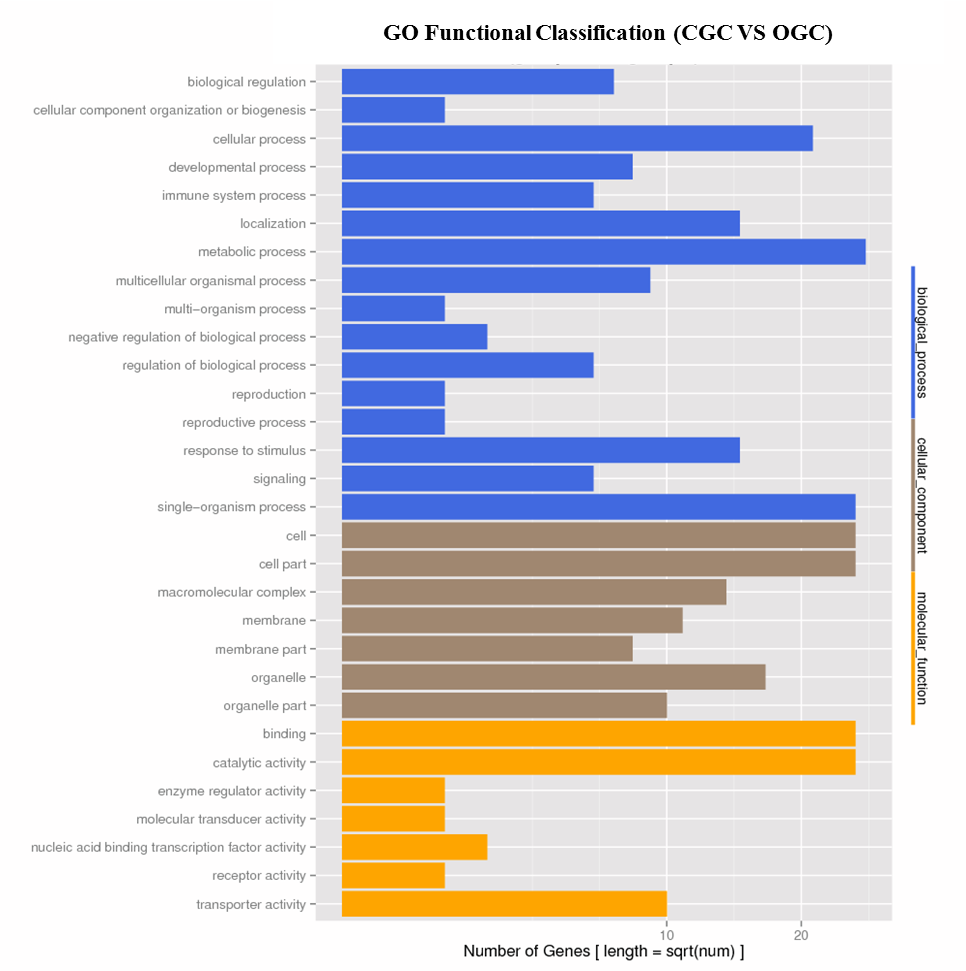
DEGs are assigned 160 GO term annotations, which are divided into three categories: cellular component, molecular function, and biological process.

**Figure S2 Scatter plot of the top 20 Kyoto Encyclopedia of Genes and Genomes (KEGG) enrichments of the differentially expressed genes (DEGs) of dorsal muscle in grass carp fed with commercial diets or faba beans for 120 days.**


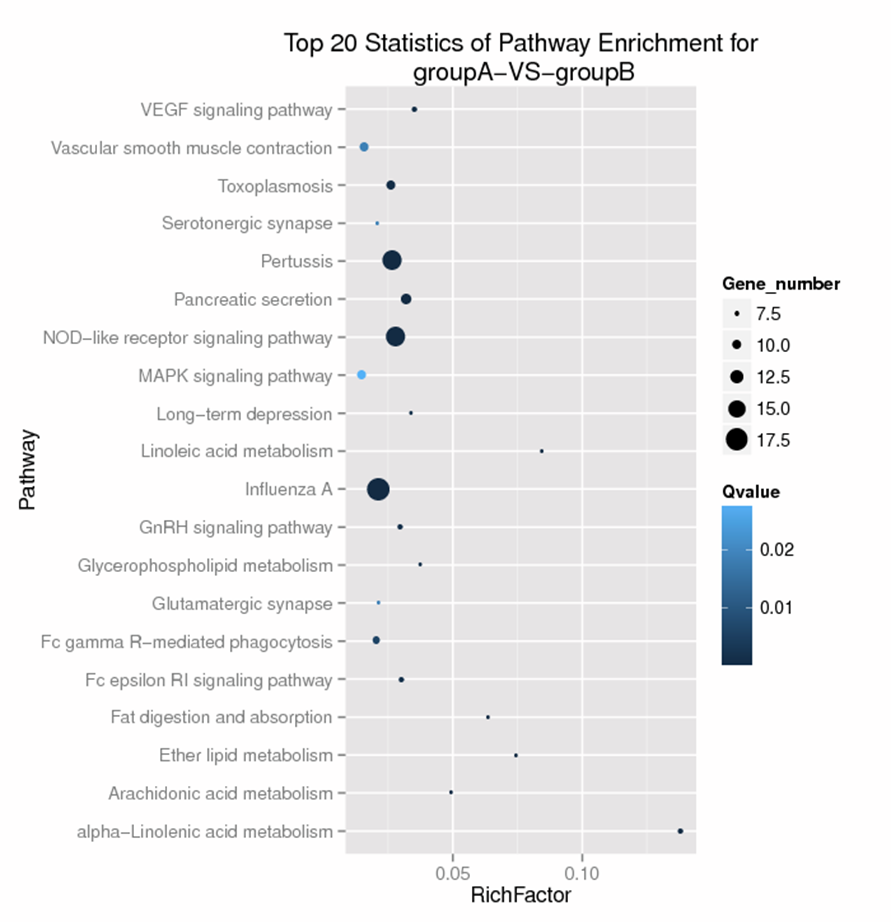
The X-axis represents the rich factor. The Y-axis is the pathway enrichment terms. Q-value represents the corrected P, and a small Q-value indicates high significance.

Table S1 Primers used in real-time quantitative PCR

| Gene | Forward (5’-3’) | Reverse (5’-3’) | Amplicon length (bp) | Amplification efficiency |
| --- | --- | --- | --- | --- |
| NRK2 | CCATCCCTTACGAGGAGTGC | GCAGCCTGCTCCTGCTATAA | 134 | 90.57% |
| AMPD | TCTCACACAGGGCTAAGGGT | GGTGAAGTTCTTCGCACAGC | 138 | 92.46% |
| Mtmr3 | TGAGGAGGATAAGCGCAGTG | TCGTGCTACACGAGAGGAAC | 81 | 90.24% |
| Myhb | TGAAGCAGAGCAGAGACGTG | GGAGTCTGGCGACGTTCTT | 116 | 94.75% |
| HBB | CCTTTGGCAACCTGTCAAGC | GCGGAATAGGTGGCCTTGAT | 112 | 93.46% |
| UCP2 | TCTGCAGGCCAGTACAACAG | GGAGGGCACAAAGCCCTTAT | 93 | 90.47% |
| GATM | CGTGGTGGGAAACGAGATCA | TTAATGAGGGGCCGATACGC | 84 | 91.64% |
| SCCT1 | GTTTATTGCTGCCGACGCAT | ACATGTTGGCACGTCAGCTA | 100 | 91.49% |
| β-actin | TCCACCTTCCAGCAGATGTGGATT | AGTTTGAGTCGGCGTGAAGTGGTA | 113 | 93.66% |
